# The Value of Adrenal Androgens for Correcting Cortisol Lateralization in Adrenal Venous Sampling in Patients with Normal Cortisol Secretion

**DOI:** 10.1155/2019/2860810

**Published:** 2019-07-31

**Authors:** Wenjing Zhang, Keying Zhu, Hongyun Li, Yan Zhang, Dalong Zhu, Xuebin Zhang, Ping Li

**Affiliations:** ^1^Department of Endocrinology, Drum Tower Hospital Affiliated to Nanjing University Medical School, Nanjing 210008, China; ^2^Department of Imaging, Drum Tower Hospital Affiliated to Nanjing University Medical School, Nanjing 210008, China

## Abstract

The management of patients with adrenocorticotropic hormone-independent Cushing's syndrome and bilateral adrenal masses is challenging. Adrenal venous sampling (AVS) has been used to identify functional lesions in previous studies, but it is not always reliable. The present study aims to address the variability of cortisol in the adrenal veins of patients without excessive cortisol secretion and investigate the use of adrenal androgens to correct the cortisol lateralization ratio in AVS. Thirty-seven patients with primary aldosteronism underwent successful AVS. Patients with normal cortisol secretion exhibited a wide range of cortisol concentrations in the right (601-89, 400 nmol/l) and left (331-35, 300 nmol/l) adrenal veins. The median cortisol gradients between adrenal venous and peripheral venous samples were 15.25 and 10.14 in the right and left sides, respectively, and the cortisol lateralization ratio (high side to low side) was as high as 9.49 (median 1.54). The mean plasma levels of cortisol in the adrenal venous and peripheral venous samples decreased from t-15 to t0. Significant positive correlations were observed between the cortisol concentrations and both androstenedione and dehydroepiandrosterone concentrations in the right and left adrenal veins. After correcting for androstenedione or dehydroepiandrosterone levels, the cortisol lateralization ratio was less than 2 in most adrenal venous samples. The present study demonstrated the wide variation in cortisol concentrations in the adrenal veins of patients with normal cortisol secretion. The adrenal androgens might be ideal analytes used as normalizers when assessing the cortisol lateralization of AVS in normal or hypercortisolism cases.

## 1. Introduction

Adrenocorticotropic hormone (ACTH)-independent Cushing's syndrome (CS) is occasionally caused by bilateral adrenocortical lesions. Such patients may have a unilateral cortisol-secreting adenoma with a contralateral nonfunctioning cortical adenoma, bilateral cortisol-secreting adenomas, or bilateral ACTH-independent macronodular adrenal hyperplasia (AIMAH) mimicking bilateral single adenomas [1]. Compared to the management of adrenal CS in patients with a unilateral cortical adenoma, management of ACTH-independent CS in patients with bilateral adrenal masses is problematic [2]. It is critical during treatment decision making to differentiate between a functioning or nonfunctioning adrenal cortical mass and to distinguish the unilateral overproduction of cortisol from bilateral disease. 

Adrenal venous sampling (AVS) has been successfully used to lateralize the source of aldosterone hypersecretion in patients with primary aldosteronism [3, 4]. Several previous studies have reported using AVS for evaluation of cortisol-producing adrenal masses [1, 5–7]. Although it is helpful in some cases, this procedure is not successful in all cases [8–10]. Thus, it is still necessary to validate the protocol and identify the underlying factors that affect the interpretation of the results.

According to the literature and our experience using AVS in primary aldosteronism (PA) cases, the following factors might interfere with the assessment of lateralization in ACTH-independent CS: (1) a stress reaction involving increased cortisol release, (2) fluctuating levels of cortisol induced by ACTH secretion, and (3) different dilutional effects in the right and left adrenals due to the adrenal venous (AV) anatomy [11]. However, few studies have addressed the effect of cortisol variation in normal AV samples [12, 13]. Furthermore, no consensus has been reached on the optimal parameters that can be used in hypercortisolism cases to correct for the cortisol gradient between the right and left adrenals caused by the above factors.

The adrenal androgens androstenedione and dehydroepiandrosterone (DHEA) are mainly produced in the zona reticularis. Recently published studies from our group and others suggested that adrenal androgens are useful for assessing the selectivity of AVS in PA [14–16]. The present study aims to investigate the variation of cortisol in AVS in patients with normal cortisol secretion. Furthermore, we aim to determine whether adrenal androgens are useful in correcting for the side-to-side gradient of cortisol caused by physiologic factors or anatomic differences.

## 2. Subjects and Methods

### 2.1. Subjects

We consecutively recruited PA patients undergoing AVS among patients referred to the endocrinology department at our hospital for suspected secondary hypertension. The diagnosis of PA was confirmed by an intravenous saline infusion or a captopril test. A 1 mg dexamethasone suppression test (DST) was performed to exclude autonomous cortisol secretion, indicated by a post-DST cortisol level less than 50 nmol/l. We performed twenty-four-hour urinary catecholamine measurement to exclude pheochromocytoma. Patients were offered AVS according to the guidelines of the US Endocrine Society [17]. Informed written consent was obtained from each participant. All procedures were performed in accordance with the principles of the Declaration of Helsinki and institutional guidelines.

### 2.2. AVS Procedure

AVS was performed as described previously [15]. The procedure was conducted between 0800 and 1200 hours by one radiologist using a bilateral simultaneous technique without cosyntropin stimulation. Blood was collected by gravity or with gentle negative pressure. Intraprocedural plasma cortisol concentration (PCC) measurement was performed to confirm correct catheter placement during AVS. In 17 patients, blood samples were collected twice at baseline, with a 15 min interval between time -15 and 0 (t-15 and t0). During this interval, the catheter remained in the adrenal vein on both sides. Heparin was injected intravenously to avoid the risk of thrombosis. Another portion of the sample was stored at 80°C for later measurement of adrenal androgens. Finally, 37 consecutive patients who underwent a total of 54 successful AVS procedures were included.

### 2.3. Measurements of Cortisol, Androstenedione, and DHEA

The PCC was measured with a commercially available kit (Immulite 2000 Cortisol, Siemens Healthcare Diagnostics Products Limited, Gwynedd LL55 4EL, United Kingdom). The intra- and interassay coefficients of variation (CVs) for PCCs were 4.6% and 6.8%, respectively. The plasma concentrations of androstenedione and DHEA were measured using a commercial ELISA kit (DRG International, Inc., USA). The intra-assay CVs for androstenedione ranged from 0.35% for high plasma concentrations to 1.50% for low concentrations. The interassay CVs for DHEA ranged from 0.71% for high plasma concentrations to 2.85% for low concentrations. The intra-assay and interassay CVs for this assay were 5.2% and 9.8%, respectively.

### 2.4. Statistical Analysis

Data are expressed as the means and SDs or, in the case of skewed distributions, as medians and ranges. Kruskal-Wallis and Mann-Whitney U tests were used to assess the significance of differences in variables at the three sampling sites or between groups. Relationships among cortisol, androstenedione, and DHEA were assessed by one-tailed Spearman's correlation coefficient (r_s_). A paired t test was used to compare the log-transformed values obtained at t-15 and t0. A value of P < 0.05 was considered significant [15]. SPSS 18.0 for Windows and GraphPad Prism 4.0 were used for the analysis.

## 3. Results

### 3.1. AV Cortisol and Adrenal Androgen Levels and Cortisol Lateralization

Patients with normal cortisol secretion exhibited a wide range of cortisol concentrations in the right (601-89, 400 nmol/l) and left (331-35, 300 nmol/l) adrenal veins. The median cortisol gradients between AV and peripheral venous (PV) samples were 15.25 and 10.14 on the right and left sides, respectively. Consistent with the cortisol concentrations, considerably higher plasma androstenedione and DHEA concentrations were detected in the right and left AV samples than in PV samples (P < 0.01). The androstenedione and DHEA gradients between AV and PV samples were approximately 2-3 times higher than those of cortisol. Although no significant difference was observed in cortisol levels between the right and left adrenal veins, the cortisol lateralization (high-side to low-side) was as high as 9.49 (median 1.54). Similar findings were also observed for androstenedione and DHEA (Table 1).

### 3.2. Variation in Plasma Cortisol Levels over Time

To investigate the variation of hormones over time, we measured cortisol levels in repeated samples from 17 PA patients obtained at 15 min intervals as described previously [15]. The mean plasma levels of cortisol in the AV and PV samples decreased from t-15 to t0. Compared to t-15, the AV cortisol levels on the right side were significantly decreased at t0 [P < 0.05, 7860 (4260-11, 360) nmol/l vs. 3920 (1515-9120) nmol/l]. However, cortisol concentrations in the left AV samples exhibited a nonsignificant decrease from t-15 to t0 [5600 (2780-9945) nmol/l vs. 3420 (1632-7665) nmol/l]. The plasma cortisol levels in the left and right AV samples decreased by 61% and 64%, respectively. As a result, the cortisol gradients between the AV and PV samples also decreased from t-15 to t0. The cortisol lateralization did not change significantly from t-15 to t0 (1.69 vs. 1.51) (Table 2).

### 3.3. Correlations between Cortisol and Adrenal Androgens

**Figure 1:**
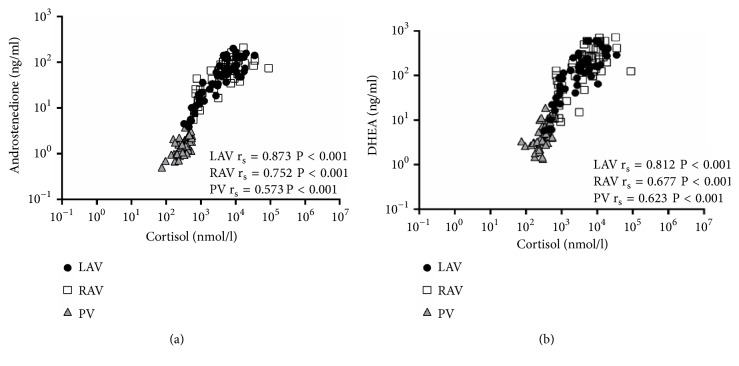
Correlations among plasma androstenedione (a), plasma DHEA (b), and plasma cortisol levels for AVS. LAV, left adrenal vein; RAV, right adrenal vein; and PV, peripheral vein.

Significant positive correlations were observed between the cortisol concentrations and both androstenedione and DHEA concentrations in the right (r_s_ = 0.752 and 0.677, P < 0.001, resp.) and left (r_s_ = 0.873 and 0.812, P < 0.001, resp.) AV samples (Figures 1(a) and 1(b)).

### 3.4. Cortisol Lateralization Ratio after Correction for Adrenal Androgens

**Figure 2:**
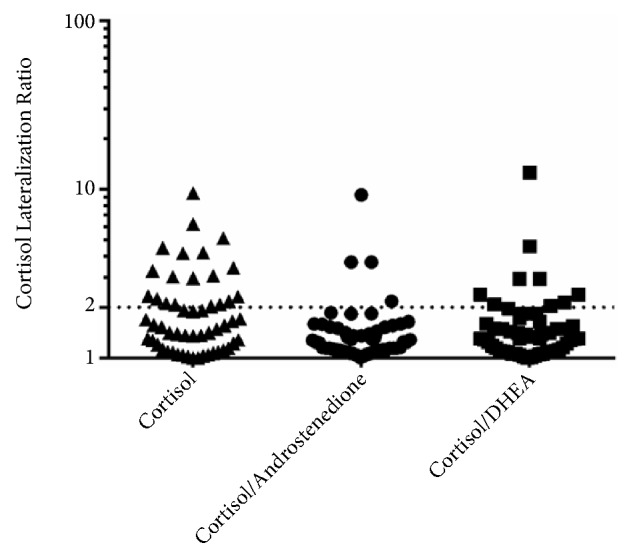
High-side to low-side adrenal vein cortisol concentration ratios with or without adrenal androgens correction. AV, adrenal vein.

Among 54 AVS procedures, 35 AVS procedures exhibited a cortisol lateralization ratio (high-side to low-side) less than 2 between the right and left AV samples, and 19 AVS procedures had a ratio greater than 2. After correction for androstenedione levels, only 4 AVS procedures had a lateralization ratio greater than 2. After correction for DHEA, 9 AVS procedures had a lateralization ratio greater than 2 (Figure 2).

## 4. Discussion

These data from patients without excessive cortisol secretion undergoing AVS demonstrated that adrenal vein cortisol concentrations exhibit great variation, including significant variation in the plasma cortisol levels over time and the cortisol gradient between the right and left adrenal veins. Furthermore, we confirmed that the adrenal androgens androstenedione and DHEA are useful in correcting for the side-to-side gradient of cortisol.

The optimum surgical treatment of ACTH-independent CS and subclinical CS is less clear when a patient appears to have bilateral adrenal cortical adenomas [2]. Bilateral adrenalectomy can resolve the condition but results in postoperative adrenal insufficiency, necessitating lifelong glucocorticoid and mineralocorticoid replacement [1, 2]. Therefore, it is necessary to differentiate among bilateral cortisol-secreting adenomas, unilateral cortisol-secreting adenoma, and nonfunctional adenomas and to identify the lesion to resect.

AVS is commonly used to distinguish the source of hormonal production in patients with primary hyperaldosteronism [3]. Several studies have reported the use of AVS for CS with bilateral masses, of which the most well-known study was conducted by the Mayo Clinic (Rochester, MN, USA) [18]. Young reported ten patients with bilateral adrenal masses and ACTH-independent CS or subclinical CS who underwent AVS. According to the results, a cortisol AV: PV gradient of 6.5 was consistent with a cortisol-secreting adenoma. If the ratio was ≤ 3.3, the lesion was considered a nonfunctional adenoma, and when the bilateral adrenal vein lateralization ratio was ≤ 2.0, the condition was considered bilateral cortisol hypersecretion [18].

Subsequent studies followed the criteria of Young in interpreting the results of AVS in patients with bilateral adrenal masses and ACTH-independent CS. However, AVS has not always been reliable in differentiating the source of excessive cortisol secretion according to literature reported from different centres [8–10]. Based on the data reported herein, the cortisol AV: PV ratio was far greater than 6.5, and the ratio changed dramatically over time in patients without excessive cortisol secretion. Furthermore, the cortisol ratio between the high and low sides was greater than 2 in half of the AVS patients with normal cortisol secretion. Therefore, two factors might interfere with the validity of AVS in adrenal-dependent CS with bilateral adrenal masses: (1) the variation of cortisol in AVS induced by stress or fluctuation of ACTH and (2) the different dilutional effect in the right and left adrenals due to AV anatomy.

To exclude the possibility of endogenous ACTH secretion interfering with the interpretation of autonomous cortisol secretion, all patients underwent AVS while receiving dexamethasone in Young's study [18]. However, not all of the studies followed this protocol, and the utility of the DST has been questioned [7, 8, 10]. It is well known that ACTH is fully suppressed in the majority of patients with ACTH-independent CS. However, it is possible that minor ACTH-independent cortisol secretion occurs from all adrenal glands, even in the setting of suppressed ACTH secretion [8]. Furthermore, dexamethasone suppression cannot eliminate the dilutional difference between the right and left adrenal veins.

The most reliable solution is to investigate novel analytes to correct for cortisol lateralization during AVS. In PA patients, “cortisol-corrected” aldosterone ratios are compared to determine whether a unilateral source of aldosterone exists. Previous studies attempted to use epinephrine or aldosterone to correct for the side-to-side cortisol gradient [7–9]. Based on the data of Freel EM et al., in patients without pheochromocytoma, a gradient in catecholamine production between the right and left adrenal glands can be a normal finding. In the case, up to 83-fold difference in epinephrine was found between the right and left adrenal veins [11]. As a result, catecholamines are of no value in correcting the cortisol gradient. Aldosterone and cortisol are both adrenal cortical hormones. However, compared to cortisol, the secretion of aldosterone is mildly regulated by ACTH. Therefore, it is predictable that the use of aldosterone might correct for the cortisol lateralization ratio caused by the dilutional effect but not by stress or fluctuation of ACTH. Thus, it is not surprising that the diagnosis by AVS in two cases proved to be incorrect in patients with bilateral adrenal masses and ACTH-independent CS [8, 9].

The adrenal androgens androstenedione and DHEA are adrenocortical products. Compared to cortisol, higher gradients of AV to PV plasma androstenedione and DHEA concentrations were detected [15, 19]. Most importantly, cortisol and adrenal androgens are both regulated by ACTH [20, 21]. As demonstrated in our study, androstenedione and DHEA closely correlated with cortisol. These merits make adrenal androgens ideal analytes to correct for cortisol variation or side-to-side dilutional differences in adrenal veins during AVS. In patients with normal cortisol secretion, the cortisol lateralization ratio caused by variation or dilutional differences almost disappeared after correction with androstenedione and DHEA.

## 5. Additional Points

*Study Limitations.* There are several limitations of the present study as follows. (1) The present study did not investigate the effectiveness of the DST on cortisol variation in patients without excessive cortisol secretion. It is suspected that the DST will alleviate the variation in cortisol levels in AVS induced by stress or the fluctuation in ACTH to some extent, but it has no effect on the different dilutions in the right and left adrenals due to AV anatomy. (2) The present data were obtained from patients with PA and normal cortisol secretion, and we did not provide evidence for the use of androstenedione and DHEA to correct AVS in patients with ACTH-independent CS. We recently showed the effectiveness of adrenal androgens in one case in clinical practice. Additional cases are needed to confirm the effectiveness of androstenedione and DHEA to correct for cortisol lateralization in ACTH-independent CS.

In conclusion, the present study promoted the use of the plasma adrenal androgens as normalizers to assess cortisol lateralization in AVS. This technique will improve the diagnostic accuracy of AVS in the localization of autonomous hypercortisolism in the setting of ACTH-independent CS in patients with bilateral adrenal masses.

## Figures and Tables

**Table 1:** Adrenal vein cortisol and adrenal androgen measurements in patients with normal cortisol secretion undergoing adrenal venous sampling [M (25-75%)].

Parameter	LAV (range)	RAV (range)	PV (range)	LAV/PV	RAV/PV	Lateralization ratio ^A^ (range)
Cortisol (nmol/l)	3270 ^*∗*^ (331-35300)	4695 ^*∗*^ (601-89400)	317 (75-557)	10.14 (1.57-88.21)	15.25 (2.01-206.47)	1.54 (1.00-9.49)
Androstenedione (ng/ml)	48.65 ^*∗*^ (3.87-200.00)	66.69 ^*∗*^ (5.57-210.21)	1.51 (0.50-3.76)	27.62 (2.52-169.34)	43.01 (6.84-129.30)	1.49 (1.00-14.91)
DHEA (ng/ml)	132.74 ^*∗*^ (5.81-616.43)	160.14 ^*∗*^ (9.14-722.21)	4.24 (1.34-18.99)	35.76 (1.83-291.26)	46.05 (2.60-243.67)	1.69 (1.00-22.52)

DHEA, dehydroepiandrosterone; LAV, left adrenal vein; RAV, right adrenal vein; and PV, peripheral vein.

^*∗*^ P < 0.01, vs. PV; ^A^ side-to-side (high-side to low-side) adrenal vein hormone gradient.

**Table 2:** Plasma cortisol levels in the adrenal veins measured from repeated samples (t-15, t0) during AVS [M (25-75%)].

Parameter	LAV (range)	RAV (range)	PV (range)	LAV/PV	RAV/PV	Lateralization ratio ^A^ (range)
Cortisol (nmol/l) t-15	5600 (2780-9945)	7860 (4260-11360)	309 (247-457)	18.22 (1.97-40.82)	24.39 (3.44-206.47)	1.69 (1.02-5.14)
Cortisol (nmol/l) t0	3420 (1632-7665)	3920 (1515-9120)	292 (234-391)	10.76 (2.16-88.21)	13.42 (2.01-65.50)	1.51 (1.00-6.22)
P (t-15 vs t0)	0.455	0.035	0.082	0.209	0.654	0.904
Variance ratio (t0–t-15/t-15)	0.61±0.51	0.64±0.37	0.17±0.16			

LAV, left adrenal vein; RAV, right adrenal vein; and PV, peripheral vein. ^A^ side-to-side (high-side to low-side) adrenal vein cortisol gradient.

## Data Availability

The data used to support the findings of this study are included within the article.
